# B-type natriuretic peptide as diagnostic and prognostic marker in various forms of acute coronary syndrome

**DOI:** 10.12669/pjms.38.4.4910

**Published:** 2022

**Authors:** Sidra Sadiq, Aamir Ijaz, Mirza Muhammad Dawood, Tayyaba Sadiq

**Affiliations:** 1 Sidra Sadiq, MBBS, FCPS, Pathology Department, Rehman Medical Institute, Peshawar, Pakistan; 2 Aamir Ijaz, MBBS, FCPS, Pathology Department, Rehman Medical Institute, Peshawar, Pakistan; 3 Mirza Muhammad Dawood, MBBS, FCPS, Pathology Department, Rehman Medical Institute, Peshawar, Pakistan; 4 Tayyaba Sadiq, BS MLT, Pathology Department, Rehman Medical Institute, Peshawar, Pakistan

**Keywords:** Acute myocardial infarction, Acute coronary syndrome

## Abstract

**Objectives:**

This study was conducted to evaluate the diagnostic and prognostic value of B-type natriuretic peptide (BNP) in different categories of acute coronary syndrome (ACS) patients on arrival.

**Methods:**

This cohort study included 197 patients admitted in Coronary Care Unit (CCU) of Rehman Medical Institute (RMI) Peshawar from January 2020 to June 2020. Patients were categorized in two subgroups. Subgroup-I with BNP below 100pg/mL. Subgroup-II having BNP above 100pg/mL. Samples were obtained on admission from these patients for Cardiac Troponin I (Trop-i), BNP and serum creatinine. BNP samples were analyzed on Cobas® using chemiluminescence method. Descriptive statistics were derived for age; gender and cardiac biomarkers. Receiver-operating characteristic curves (ROC) were generated. Diagnostic accuracy parameters were determined for Non ST- segment elevation myocardial infarction (STEMI), ST- segment elevation myocardial infarction (STEMI) and unstable angina (UA).

**Result:**

One hundred fourteen patients (58.2%) were males and 82 were females (41.8%).Our of this 89 patients were NSTEMI in group II. Mean BNP was 1438±1463.Age distribution shows 120 individuals were over 55 years (61.2%). Hypertension, diabetes, smoking and previous infarcts were the risk factors for ACS. Sensitivity, specificity, Positive Predictive Value (PPV), Negative Predictive value (NPV), likelihood ratios, and overall accuracy of BNP at admission for the entire sub categories in ACS (cut-off value 100 pg/mL) were determined by using Trop-i the gold standard. ROC curve showed AUC = 0.557, (95% confidence interval: 0.476–0.638). When Pearson correlation was applied, BNP was found to be a noteworthy independent predictor.

**Conclusion:**

BNP can be a useful Biomarker along with standard cardiac biomarkers in various categories of patients with ACS.

## INTRODUCTION

Ischemic heart disease (IHD) is regarded as one of the important leading causes of disability and death across the globe.^1^ Acute coronary syndrome (ACS) applies to a variety of events reporting ischemia, ranging from stable angina without myocardial necrosis to ST –segment elevation myocardial infarction (STEMI).^2^ Thrombosis and atherosclerotic plaque destruction cause ACS to grow. Aside from these two, vasoconstriction is another significant contributor to ACS.^3^ For risk stratification in patients with ACS, serial ECG monitoring and cardiac biomarker measurements are needed as part of the diagnostic workup.^4^ BNP has shown to be a good indicator of diastolic dysfunction.^5^ It is claimed to be a strong indicator of diagnosis and prognosis in ACS patients.^6^

BNP is believed to have its role as natriuretic hormone and vasodilation. Among other effects are inhibition of the Renin-Angiotensin-Aldosterone system(RAAS), and sympathetic nervous system.^7^ After an ischemic occurrence, BNP levels peak in 14 and 40 hours.One of the most frequent reasons for admission to CCU is Non ST segment- elevation Myocardial infarction (NSTEMI), which could have a number of causes.^8^ BNP levels were found to be more in stable angina than in unstable angina (UA)in another cross-sectional analysis.^9^

Despite the fact that much research has been done on the significance of BNP in Heart Failure (HF), the novelty of the current study was to assess if BNP could predict mortality, heart failure, conservative management and cardiovascular events such as atrial fibrillation in patients diagnosed with ACS at RMI’s CCU.

## METHODS

It was a cohort study conducted on 197 eligible participants aged 18 to 80 years irrespective of the gender admitted to the CCU of Rehman Medical Institute (RMI) Peshawar between January to June 2020. In our CCU, these patients are routinely managed as per a scientific clinical evaluation that includes the following guidelines:

(i) Serum Cardiac Troponin I (Trop-i) at the initial visit.
(ii) Performing an electrocardiogram (ECG); and
(iii) An echocardiography.

If none of the preceding three investigations have identified myocardial ischemia or necrosis, an exercise tolerance test (ETT) is performed.

Patients were evaluated for HF using American Heart Association (AHA) guidelines prior to blood sampling. The current study included plasma BNP, which was used in the investigation panel and acquired on initial assessment with the intention of correlating BNP with the definitive diagnosis. Study was approved by ethical review committee of RMI, Peshawar (RMI/RMI-REC/approval/63 dated: December 21, 2019). Informed consent was obtained from the patients. The data collection was performed using standard clinical practice. On arrival Plasma BNP was assayed on the same EDTA-anti coagulated blood specimen for Trop-i on Cobas®. The assay is a two-step immunoassay that uses chemiluminescent micro particle immunoassay (CMIA) for the quantitative determination of BNP. The kit’s analytic sensitivity is 5 pg/mL, with a linearity of 5000 pg/mL according to the manufacturer’s recommendations. The upper normal limit is 100 pg/mL. The recommended Quality Control (QC) requirements for BNP assay is that a single sample of each control level to be tested once every 24 hours each day of use. CV is less than 5%.

### Inclusion criteria:

Patients with or at risk of ACS, Coronary Heart Disease (CHD) and willing to give informed consent were included in the study.

### Exclusion criteria:

Patients with previous MI, ejection fraction (EF) less than 30% and patients having cardiomyopathy, renal, liver, malignant, infectious, or inflammatory diseases.

### Data Collection Procedure:

The participants were distributed into two subgroups based on the empirical cut-off value for plasma BNP. Patients in Sub Group I had plasma BNP level of less than 100pg/mL, while those in Sub Group-II had plasma BNP levels greater than 100pg/mL. The time between their admittance to the hospital and their stay in the hospital was termed as the follow-up period. The outcomes examined included conservative management, mortality, per cutaneous coronary intervention (PCI) and coronary artery bypass graft (CABG).

During their hospital stay, patients were assisted by one of research assistant who recorded all diagnostic tests and patient information on a Performa, as well as monitored final diagnosis (by a Consultant Cardiologist) and events according to pre-defined procedure descriptions. In the vast majority of cases, at least two physicians double-checked the final diagnosis:

- Trop-i above 400pg/mL in any specimen taken within the first three to six hours after admission, with presence or absence of ST/T changes on the ECG and when no other obvious causes for the chest pain, the patient was labeled as NSTEMI.
- When Trop-i was within reference values and related with ST-segment depression (0.1 mV) or T-wave inversion in the ECG, ischemia on ETT, or severe CHD on an angiography, patient was labeled as UA.
- STEMI was confirmed by ECG with ST segment elevation.
- In the CCU, if a complete diagnostic checkup revealed no myocardial necrosis or ischemia, suggesting the absence of ACS. After percutaneous Tran’s luminal Coronary Angioplasty, patients with elevated Trop-i levels were not diagnosed with AMI.

### Statistical Analysis:

Data was analyzed on SPSS version 23. Age, gender, risk factors, and cardiac biomarkers were all subjected to descriptive statistics.

ROC curves were created using Trop-i as the gold standard to compare the prognostic and diagnostic importance of BNP measurements in STEMI, NSTEMI, and UA. Sensitivities, specificities, positive predictive value (PPV), Negative predictive value (NPV), Likelihood ratio positive (LR+), likelihood ratio negative (LR-) and overall accuracy were determined for BNP in STEMI, NSTEMI and UA. Pearson correlation was used to create a predictive association between BNP levels and ACS diagnosis, which was then adjusted for clinical and laboratory data. A *p*-value of 0.05 was found to be statistically acceptable in all tests.

## RESULTS

A total of 196 patients were included in this study out of which male were 114(58.2%). Majority of the patients 120(61.2%) were in the age group above 55 years. Hypertension was the leading risk factor in patients 47(23.9%) (Table-I).

**Table-I:** Study Participants’ Demographic Characteristics: n=196.

Variables	f (%)
***Gender***	
Male	114(58.2)
Female	82(41.8)
***Age group***	
25-40 years	26(13.3)
41-55 years	50(25.5)
>55 years	120(61.2)
***Risk factors***	
Diabetes	39(19.9)
Hypertension	47(23.9)
Smoking	18(9.2)
Previous infarct	14(7.1)

The initial ECG findings showed that most of the patients had ST depression 81(41.3%) followed by T wave 70(35.7%). Regarding diagnosis in our patient’s majority had NSTEMI 98(50.0%), and most of the patients were managed conservatively 89(45.4%). Rests of the parameters. Table-II.

**Table-II:** Clinical status of the participants: n=196.

Variables	f(%)
***ECG findings***	
ST elevation	45(23.0)
ST depression	81(41.3)
T wave	70(35.7)
***Diagnosis***	
NSTEMI	98(50.0)
STEMI	42(21.4)
Unstable angina	56(28.6)
Troponin ng/ml(mean/SD)	4.89±4.47
BNP pg/ml(mean/SD)	1438.35±1463.60
***Outcomes***	
Conservative management	89(45.4)
PCI	49(25.0)
CABG	29(14.8)
DEATH	29(14.8)

Age, gender, ST-segment depression or elevation or T wave inversion on admission, and diagnosis of STEMI, NSTEMI, and UA on index hospitalization were all directly associated with elevated BNP levels (>100 pg/ml) (p value 0.001). The correlation of BNP with clinical and laboratory results shown in Table-III.

**Table-III:** Age, gender, and clinical correlation with BNP level.

Variable	BNP level		P value

	<100	>100	
***Gender***			
Male	27	87	0.303
Female	16	66	
***Age group***			
25-40 years	9	17	0.176
41-55 years	8	42	
>55 years	26	94	
***ECG changes***			
ST depression	18	63	<0.001
ST elevation	12	33	
T wave	13	57	
***Diagnosis***			
NSTEMI	9	89	<0.001
STEMI	9	33	
Unstable angina	7	49	

### Diagnostic Accuracy of BNP:

**Fig.1:**
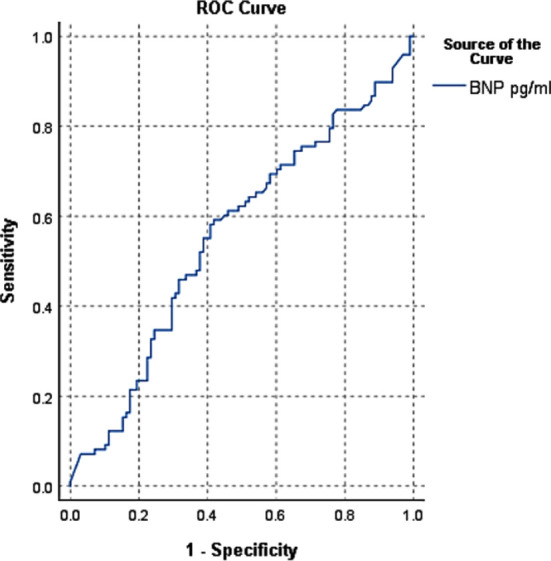
ROC curve for NSTEMI.

BNP had the best predictive cut-off value of 100 pg/mL. (Area under the curve (AUC) = 0.557, 95% CI = 0.476 – 0.638), according to ROC curve review. Sensitivity, specificity, PPV, NPV, LR+, LR-, overall accuracy for NSTEMI were 100%, 98.84%, 90.54%, 87.24%, 1.02, 0.99and 90.8% respectively (Fig.1). Sensitivity, specificity, PPV, NPV**,** LR+, LR- of BNP - 100 pg/mL for the diagnosis of ACS are illustrated in Table-IV.

**Table-IV:** Diagnostic accuracy of BNP for Various forms of acute coronary Syndrome (ACS).

Diagnosis	Sensitivity	Specificity	PPV	NPV	LR+	LR-	Overall Accuracy
NSTEMI	100%	98.84%	90.54%	87.24%	1.02	0.99	90.8%
STEMI	100%	100%	95.24%	100%	1.01	1.01	78.5%
UA	86.24%	76.98%	89.54%	79.75%	1.13	0.90	87.5%

**Fig.2:**
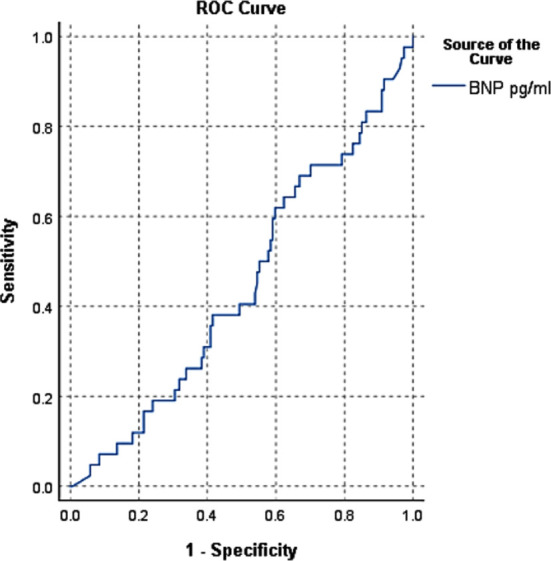
ROC curve for STEMI.

Sensitivity, specificity, PPV, NPV, LR+, LR- and overall diagnostic accuracy STEMI were 100%, 100%, 95.24%,100%,1.01,1.01 and 78.5% respectively (Fig.2).

**Fig.3:**
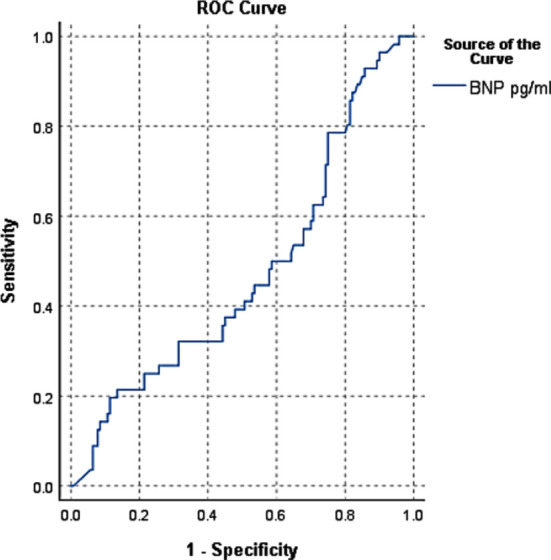
ROC curve for unstable angina.

For UA its sensitivity was 86.24%, specificity was 76.98%, PPV, 89.54%, NPV 79.75%, LR + 1.13,LR- 0.90, overall diagnostic accuracy 87.5%(Fig.3).

## DISCUSSION

Since the year 2000, clinical assessment risk scores like Thrombolysis in Myocardial Infarction (TIMI) and Global Registry of Acute Coronary Events Score 17(GRACE 17) risk scores have been used to assess the probability of short and long term cardiovascular outcomes in patients with ACS since they’re relatively simple to figure out at the bedside. Unfortunately, for longer follow-ups the accuracy of these risk factor aggregates is inadequate. Researchers are searching for new and more accurate indicators of mortality prediction as a result of this experience. According to new research, plasma BNP concentrations in patients with ACS who arrive in the CCU with chest pain are higher in those who have NSTEMI than in those who have UA or STEMI. Shon et al published a 14,887-patient study with similar results.^10^ Takahashi et al had similar findings.^11^ The underlying pathological mechanism was unclear, but it was suggested that ischemic cardiomyocytes release BNP directly, in response to myocardial injury caused by increased ventricular wall stress. BNP levels contributed significantly to diagnostic efficiency for NSTEMI when assessed in conjunction with these necrosis markers on admission, raising the sensitivity and NPV to 100% and 87.24%, respectively, as shown in Fig.1. Wong et al published a report that back up their research.^12^,^13^ In individuals with normal Trop-i (400 pg/mL) on arrival, risk of AMI is greater in patients with a BNP greater than 100 pg/mL. For STEMI and UA, BNP established high sensitivity and specificity, as well as PPV and NPV as Shown in Fig. 2 and 3.

BNP levels were assessed hours after admission to the hospital in some trials. A study conducted on 88 patients of ACS patients admitted to a CCU and discovered a noteworthy increase in the degree of ACS diagnosis as BNP levels increased.^14^ In this analysis, BNP analysis done on admission provides important prognostic information. Xu et al suggested the role of BNP elevation in ACS prognosis.^15^ These results corroborate our own. In previous researches, BNP was found to be a reliable and self-determining prognostic marker in subjects with all categories of ACS^16^-^18^ when assessed upon arrival at the hospital.

BNP levels rises soon after cardiac ischemia due to stress, according to several reports.^19^ The results not only demonstrate the connection between BNP and pathogenesis of ACS, but also suggest that BNP may have diagnostic and prognostic value in patients with MI who present to the CCU as evident from the work of Arafath et al.^20^,^21^ Similar findings are suggestive from the work of Noureen et al.^22^

Sivachandran et al. selected a sample group that mostly consisted of older people, with a higher male population, which is consistent with our results.^23^ In our research, we discovered a connection between increasing BNP levels and diagnosis. The results were important in all three subgroups of patients (*p*value0.001). These results are consistent with our findings.^24^-^26^

In another study of 926 patients conducted by Madmoli et al. diabetes and hypertension were the leading risk factors for ACS. This result matched what we found in our research.^27^ Further support is provided by Hoo et al.^28^

The results of this study corroborate previous studies and add to our understanding of increasing BNP in patients with acute cardiac ischemia, as well as its pathogenesis. Next, the incremental rise in BNP levels observed in these patient subgroups appears to endorse the biological gradient of myocardial hypoxic conditions in ACS—where ACS indicates a greater ischemic burden than UA. Secondly, a short-term rise in BNP does not seem to be attributed to MI. BNP, on the other hand, tends to be a predictor of ischemic strain, which results in diastolic dysfunction. As a result, BNP along with ECG and troponin blood levels is a good predictor of ACS.

### Limitations of the study:

There are some drawbacks to the current research. There was no comprehensive assessment of Left ventricular (LV) function. In ACS patients, LV function is widely recognized as one of the most significant predictors of outcome.^29^ According to previous research, BNP is a stronger indicator of outcome. As a result, a comparison of the LV ejection fraction calculated by echocardiography with BNP calculation in the current study would have been useful.

BNP was only assessed for the first 6 to 24 hours of admissions, which is another limitation. As obvious from various studies BNP level begins to rise 24 hours after AMI.^16^ As a result, our research does not provide any insight into the significance of consecutive BNP measurements in ACS evaluation. Finally, long-term assessment on the performance of BNP in ACS patients would have provided more prognostic and diagnostic information.^30^

## CONCLUSIONS

Plasma BNP can be used as an early indicator in ACS patients and its use in the CCU in combination with ECG and troponin should be considered in assessment of patients with cardiac infarction risk.

### Authors’ Contribution:

**SS** conceived, designed and Manuscript writing.

**SS, MMD and TS** did data collection and statistical analysis

**AI** Editing,, review and final approval of manuscript and he is responsible and accountable for the accuracy or integrity of the work.
